# The impact of intensive care unit diaries on patients’ and relatives’ outcomes: a systematic review and meta-analysis

**DOI:** 10.1186/s13054-019-2678-0

**Published:** 2019-12-16

**Authors:** Bruna Brandao Barreto, Mariana Luz, Marcos Nogueira de Oliveira Rios, Antonio Alberto Lopes, Dimitri Gusmao-Flores

**Affiliations:** 1 Intensive Care Unit, Hospital da Mulher, Rua Barão de Cotegipe, 1153, Roma, Salvador, Bahia 40411-900 Brazil; 2 Núcleo de Epidemiologia Clínica e Medicina Baseada em Evidências, Hospital Universitário Professor Edgard Santos, Universidade Federal da Bahia, Salvador, Brazil; 3 Departamento de Medicina Interna e Apoio Diagnóstico, Faculdade de Medicina da Bahia, Universidade Federal da Bahia, Salvador, Brazil; 4 Programa de Pós-Graduação em Medicina e Saúde, Faculdade de Medicina da Bahia, Universidade Federal da Bahia, Salvador, Bahia Brazil; 5 Intensive Care Unit, Hospital da Cidade, Salvador, Bahia Brazil

**Keywords:** ICU diary, Post-traumatic stress disorder, Anxiety, Depression, Quality of life

## Abstract

**Background:**

Memory gaps in intensive care unit (ICU) survivors are associated with psychiatric disorders. The ICU diaries improve the patient’s factual memory of the ICU, but it is not clear if they reduce the incidence of psychiatric disorders in patients and relatives after hospital discharge. The aim of this study is to evaluate the literature on the effect of ICU diaries for patients admitted in ICU and their relatives.

**Methods:**

Two authors independently searched the online databases PubMed, OVID, Embase, EBSCO host, and PsycINFO from inception to July 2019. Studies were included if the intervention group (ICU diary) was compared with a group with no diaries and the sample was comprised patients ≥ 18 years old admitted in the ICU for more than 24 h and their relatives. Randomized clinical trials, observational studies, letter with original data, and abstracts were included, irrespective of the language. The search was not limited by any specific outcome. Review articles, commentaries, editorials, and studies without a control group were excluded. Structured tools were used to assess the methodological quality (“Risk Of Bias In Non-randomized Studies of Interventions (ROBINS-I)” for cohort studies and the “Cochrane Risk of Bias tool” for included RCTs and before/after studies). A random-effects model was employed considering the anticipated variability between the studies.

**Results:**

Seven hundred eighty-five titles were identified for screening. Two additional studies were selected after a reference search, and after a full-text review, a total of 12 studies were included. When pooling the results, ICU diary was associated with lower risk of depression (RR 0.41, 95% CI 0.23–0.75) and better quality of life (10.3 points higher in SF-36 general health score, 95% CI 0.79–19.8), without a decrease in anxiety or post-traumatic stress disorder (PTSD). For the relatives receiving an ICU diary, there was no difference in the incidence of PTSD, anxiety, or depression.

**Conclusion and relevance:**

This systematic review and meta-analysis supports the use of ICU diaries to reduce the risk of depression and preserve the quality of life of patients after ICU admission. ICU diaries do not seem to have any beneficial effect on the relatives of the patients.

**Trial registration:**

PROSPERO, CRD42019136639

## Introduction

Patients surviving the intensive care unit (ICU) frequently experience memory gaps and unpleasant recall after ICU discharge [1]. These memory changes are often associated with the development of symptoms of anxiety, depression, and post-traumatic disorder [1] that impacts negatively on the health-related quality of life [2–5]. The need to cope with negative emotions and symptoms, the presence of *delirium*, in addition to periods under sedation and sleep deprivation during critical illness are factors that may predispose to these phenomena [6].

In 1999, Backman and Walther published what seems to be the first report of the use of ICU diaries as a way to concretize what happened to the patients during ICU care and help them to understand the chain of events during the ICU stay [7]. The use of ICU diaries has been proposed as a tool to help fill the patient’s memory and reduce the incidence of psychiatric disorders in patients and their relatives after ICU discharge.

Clinical trials have been conducted in order to evaluate the impact of ICU diaries on the development of psychiatric disorders and on quality of life [8, 9], and a meta-analysis summarized these findings suggesting a potential benefit with these approaches [10]. Since the publication of this meta-analysis, new studies were published, including three randomized trials [11–13].

The aim of this study is to reevaluate the current literature on the effect of ICU diaries for patients admitted in the ICU and their relatives, giving also a detailed description of the diary structure, healthcare impression on writing diaries, and patients’ feedbacks on receiving an ICU diary.

## Methods

Preferred Reporting Items for Systematic reviews and Meta-Analysis (PRISMA) guidelines were used in the preparation of this review (the checklist can be found in Additional file 1). The protocol was prospectively published on the International Prospective Register of Systematic Reviews (PROSPERO)—CRD42019136639.

### Search strategy

Two authors (BBB, ML) independently searched the online databases PubMed, OVID, Embase, EBSCO host, and PsycINFO from inception to July 2019 using Medical Subject Headings with search terms aiming to capture those publications which contained variations of “intensive care” and “ICU diary.” Full search strategies can be found in Additional file 2.

This search was supplemented by reviewing all references of relevant articles and searches in Google Scholar. The automatic alert system of PubMed was used to identify studies published during the process of the analysis of the results and writing of the manuscript. Any disagreement was solved by a discussion or review by a third author (DG-F).

### Study selection

Articles were included if they were original research where an ICU diary intervention group was compared with a group without diaries. Randomized controlled trials (RCT), prospective or retrospective cohort, before-and-after study, letter with original data, case-control studies, and abstracts of congress were all included, irrespective of the language in which the study was written. The studies were included regardless of the outcomes studied. If the data needed could not be extracted, an e-mail was sent to the authors in order to obtain the information. Studies with samples of adult patients (≥ 18 years old) admitted to an ICU for more than 24 h and their family members were eligible for the review.

Studies were excluded if they were review articles, commentaries, and editorials or when the key results were not found even after attempting contact with the authors.

### Data extraction

A structured template was used for the extraction of data from the included studies. Data collected included study design, location, publication year, length of follow-up, and study-specific outcomes assessed with respective scales and cutoffs used (when appropriate). These data were independently extracted by three authors (BBB, ML, MNR) and compared for concordance.

For pooling the results, data from individual studies were extracted as dichotomous outcomes and as means, difference of means, or median when appropriate. The latter format was extracted because important outcomes, such as quality of life or intensity of psychological symptoms (e.g., anxiety, depression, and post-traumatic stress symptoms), can only be measured as mean or median using appropriate scales or scores.

Due to the difference of the time of follow-up (with some studies having multiple time points), the outcomes regarding the first follow-up after the intervention were extracted.

### Risk of bias

Structured tools were used to assess the methodological quality of the included studies. This included the Risk Of Bias In Non-randomized Studies of Intervention (ROBINS-I) [14, 15] for cohort studies and the “Cochrane Risk of Bias tool” [15] for included RCTs and before-and-after studies. Three authors (BBB, ML, MNR) independently assessed the risk of bias of the included studies, and any disagreement was solved by a discussion or a review by a fourth author (DG-F). The ROBINS-I tool is based on the Cochrane Risk of Bias tool for randomized trials, in which risk of bias is assessed within specified bias domains (confounding, selection of participants into the study, classification of interventions, deviations from intended interventions, missing data, measurement of outcomes, selection of the reported result). For the “Cochrane Risk of Bias tool,” a study summarized as with a high risk of bias was judged to have a “high risk of bias” for one or more key domains.

### Statistical analysis

Data analysis was performed using the “meta” and “metafor” packages of the R software [16] version 3.5.1. Risk ratios (RRs) and 95% confidence intervals (CIs) were used, as summary statistics for studies with binary outcomes and means for studies with continuous outcomes. The studies with binary outcomes were integrated with the Mantel-Haenszel method, and for studies with continuous variables, the inverse-variance method was used for calculating the weighted average. We used the Hartung-Knapp adjustment for random-effects model [17] and the Sidik-Jonkman estimator for tau^2^. The Hartung-Knapp-Sidik-Jonkman method was chosen for random-effects meta-analysis because it outperforms the DerSimonian and Laird approach when the number of studies included in the meta-analysis is small (less than 20 studies), even when studies of different sizes are combined [18, 19]. Statistical heterogeneity was quantified with the *I*^2^ statistics. A random-effects model was employed considering the anticipated variability between the trials regarding patient samples and medical interventions. The 95% prediction interval was also calculated [20, 21] to estimate where the true effects are to be expected for 95% of studies that might be conducted in the future. A two-tailed *p* value of less than 0.05 was considered statistically significant.

For studies in which the mean value was presented without standard deviation, we estimated the standard deviation using the *p* value and group sample size to calculate the *t* value using the RevMan Calculator available at the Cochrane Training website [22]. For studies in which data were presented as the median and interquartile range (IQR), we estimated the mean and standard deviation from these values [23–25]. Funnel plots and Egger test were used to test for detecting small-study effects. The R script is available in Additional file 3.

Those studies that reported outcomes that could not be pooled, because they were unique to that particular study, were presented in a qualitative analysis.

## Results

**Fig. 1:**
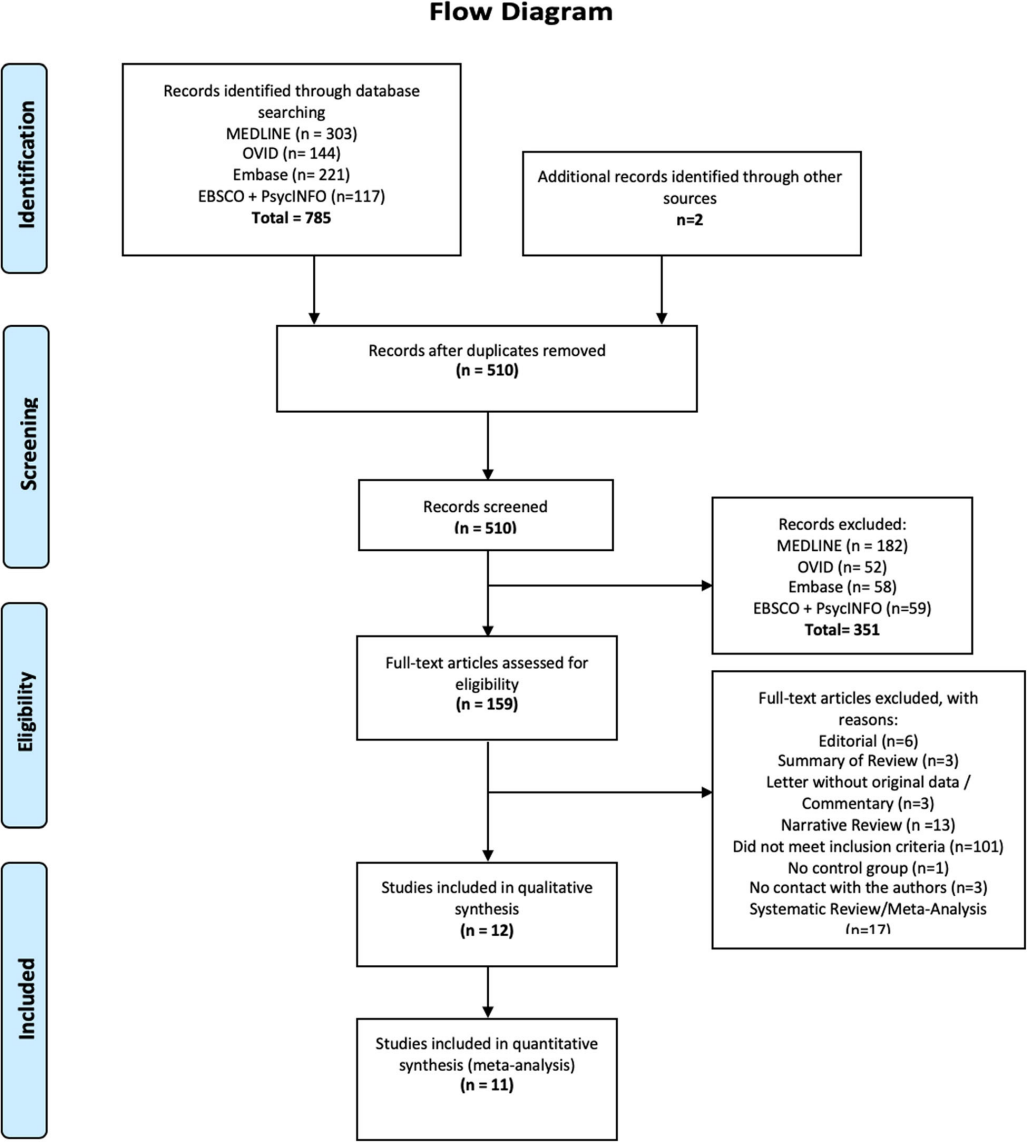
Flowchart of the systematic review

We identified 785 individual titles and abstracts for screening. One hundred fifty-nine were selected for a full-text review of which 10 satisfied all inclusion and exclusion criteria [8, 9, 11, 13, 26–31]. Two additional studies were included following a search of references [12, 32], bringing the total number of included studies to 12. Figure 1 outlines the flowchart of the search, with respective reasons for study exclusion. Of the 12 included trials, 6 were RCTs [8, 9, 11–13, 30], 2 were before-and-after studies [28, 32], and the remaining 4 being an observational cohort design [26, 27, 29, 31]. The measurement of agreement of study selection is presented in Additional file 2. There was no disagreement on data extraction. Characteristics of the included studies are summarized in Table 1, and the structure of the diaries used in the studies is presented in Additional file 2.

**Table 1:** Characteristics of studies included in the meta-analysis

Author	Year	Design	Subjects	Sample size	Country	Diary delivery	Follow-up	Tools	Risk of bias
Garrouste-Orgeas et al. [13]	2019	RCT	Patients and relatives	657	France	ICU discharge	90 days after ICU discharge	HADS, IES-R	High
Nielsen et al. [12]	2019	RCT	Patients and relatives	116	Western Denmark	ICU discharge	90 days after ICU discharge	HADS, PTSS-14, SF-36	High
Kredentser et al. [11]	2018	RCT	Patients	58	Canada	30 days after ICU discharge	90 days after ICU discharge	HADS, IES-R	High
Jones et al. [30]	2012	RCT	Relatives	36	Sweden and the UK	30 days after ICU discharge	90 days after ICU discharge	PTSS-14	High
Jones et al. [8]	2010	RCT	Patients	352	Denmark, Italy, Norway, Portugal, Sweden, and the UK	30 days after ICU discharge	90 days after ICU discharge	PDS, PTSS-14	High
Knowles et al. [9]	2009	RCT	Patients	36	UK	30 days after ICU discharge	3 weeks	HADS	High
Fukuda et al. [32]	2015	Before-and-after	Patients	40	Japan	1 week after ICU discharge	10 days after the post-ICU survey and prior to the discharge from the hospital	HADS, ASDS	High
Garrouste-Orgeas et al. [28]	2012	Before-and-after	Patients and relatives	143	France	ICU discharge	3 months (HADS/PDEQ), 12 months (IES-R)	HADS, PDEQ, IES-R	High
Åkerman and Langius-Eklof [26]	2018	Obs	Patients	419	Sweden	Unclear	2, 6, and 12 months	3-set 4P	Moderate
Glimelius Petersson et al. [29]	2015	Obs	Patients	96	Sweden	ICU discharge	2 months	PTSS-14	Serious
Svenningsen et al. [31]	2014	Obs	Patients	360	Denmark	1 week after ICU discharge	6 months	SF-36	Serious
Backman et al. [27]	2010	Obs	Patients	499	Sweden	Hospital discharge	6 months	SF-36	Moderate

*RCT* randomized controlled trial, *ICU* intensive care unit, *UK* United Kingdom, *Obs* observational cohort, *ROBINS-I* Risk Of Bias In Non-randomized Studies of Interventions, *HADS* Hospital Anxiety and Depression Scale, *PDEQ* Peritraumatic Dissociative Experiences Questionnaire, *PTSS-14* Post-traumatic Stress Symptoms screening tool, *SF-36* Modified Medical Outcomes Short Form, *IES-R* Impact of Event Scale-Revised, *ASDS* Acute Stress Disorder Scale, *PDS* Post-traumatic Stress Disorder Diagnostic Scale

### Overview of methodological quality

All RCTs and before-and-after studies were assessed as high risk of bias (the detailed evaluation is presented in Additional file 2: Figure S2). All the cohort studies were assessed as moderate or serious risk of bias according to ROBINS-I (Table 1). There was no disagreement on the final judgment of the risk of bias.

### The effect of ICU diaries on patients’ outcomes

#### Post-traumatic stress disorder

Six studies investigated the effect of ICU diaries on PTSD symptoms and diagnosis. Four of them are RCT [8, 11–13]. The scales used in the evaluation were Post-traumatic Stress Symptoms (PTSS-14) screening tool, Impact of Event Scale-Revised (IES-R), and PTSD Diagnostic Scale (PDS). Both the PTSS-14 and IES-R scales were dichotomized in order to diagnose PTSD (Additional file 2: Table S1).

##### Severity of symptoms measured by mean/median IES-R and PTSS-14

Jones et al. [8] reported a reduction in the PTSS-14 scores for those with severe symptoms of PTSD (a decrease in 23 points in PTSS-14 for those with 45 points or more, *p =* 0.04). Garrouste-Orgeas et al. [28] reported a reduction in PTSD symptoms with ICU diaries measured by mean IES-R score. The remaining studies [11–13, 29] did not find any difference in the severity of symptoms between those who received or not the ICU diary.

##### PTSD diagnosis

**Fig. 2:**
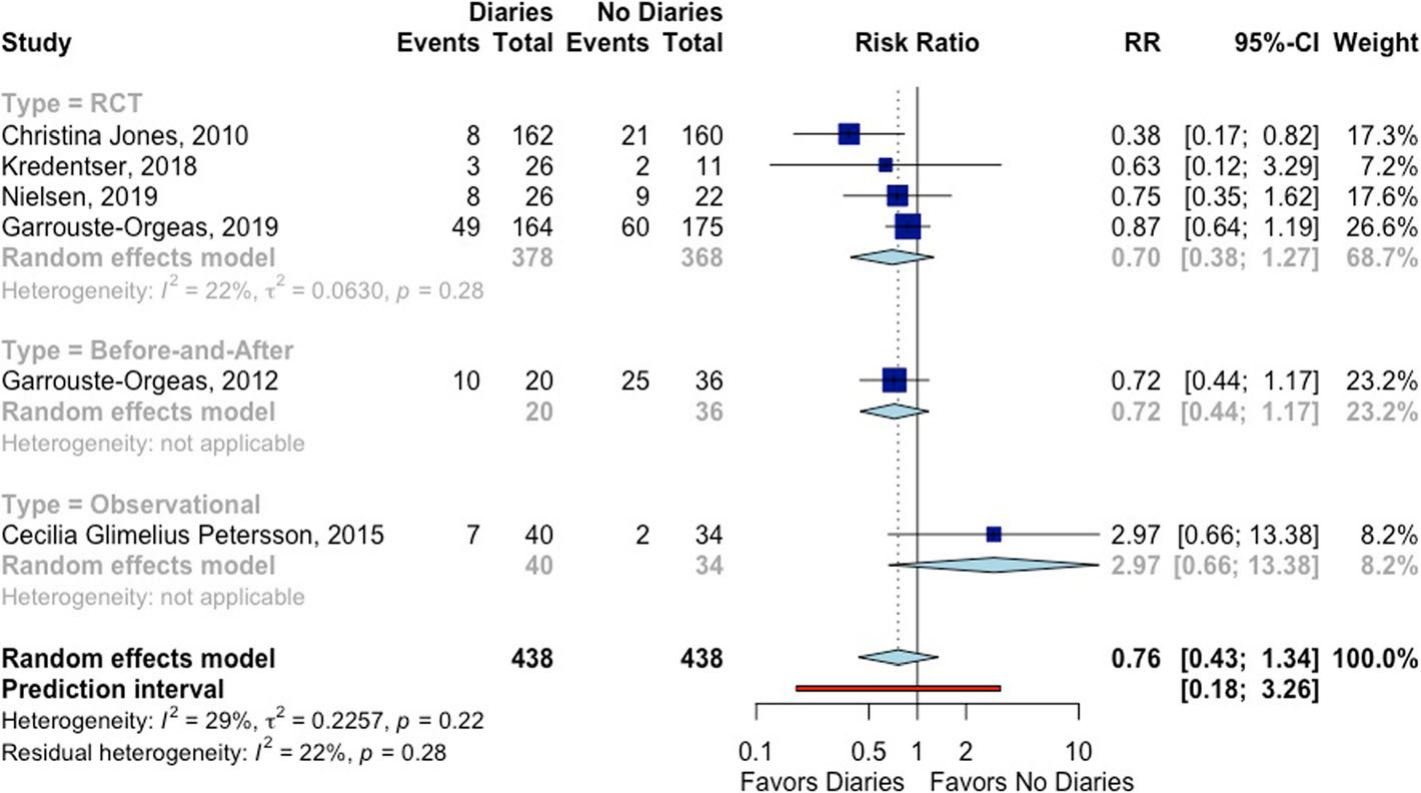
The effect of ICU diaries on post-traumatic stress disorder in patients

Jones et al. [8] reported a protective effect of ICU diaries regarding PTSD diagnosis. By contrast, the Glimelius Petersson study [29] reported an increase in PTSD cases in those who received diaries. The difference in PTSD between the groups that received and not received an ICU diary was not observed in the remaining studies [11–13, 28] (Fig. 2).

#### Depression

Six studies investigated the effect of ICU diaries on depression diagnosis and severity of symptoms [9, 11, 12, 28, 32]. Four of them are randomized controlled trials [9, 11–13]. All studies used the same scale to evaluate and diagnose depression—the Hospital Anxiety and Depression Scale (HADS)—but different cutoff points were used to dichotomize the variable (Additional file 2: Table S2).

##### Severity of depressive symptoms measured by mean/median HADS

Two RCTs reported a decrease of depressive symptoms in patients that received the ICU diary [9, 11]. Nielsen et al. [12] and Garrouste-Orgeas et al. [28] did not report HADS score as a continuous variable. Garrouste-Orgeas et al. [13] did not find any difference between the groups. Fukuda et al. [32] reported that receiving the ICU diary decreased the depressive symptoms (from 8.6 ± 5.0 to 7.2 ± 4.3 points in HADS, *p =* 0.003) only in patients with distorted memories of the ICU admission (Additional file 2: Figure S2).

##### Depression diagnosis

**Fig. 3:**
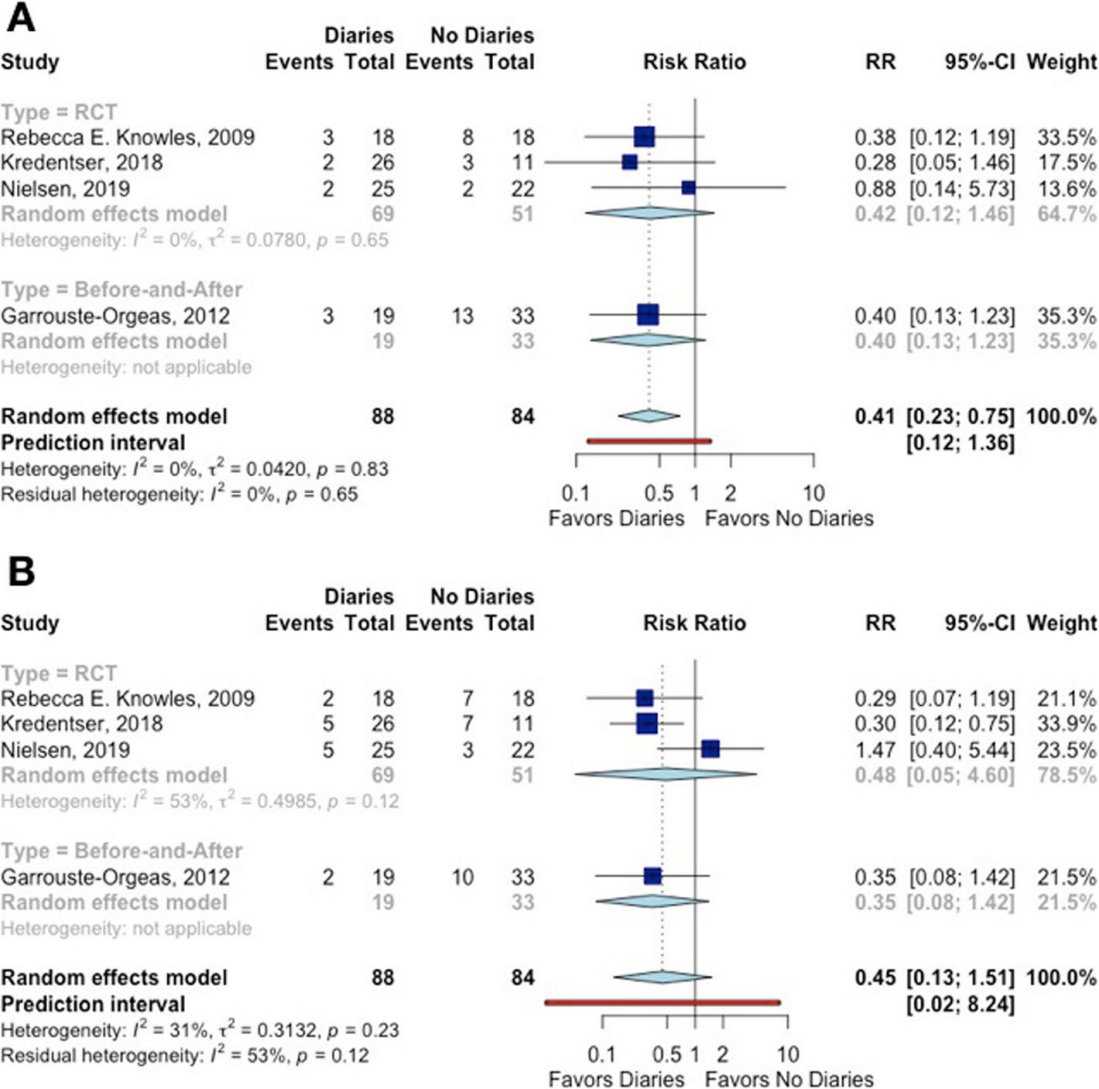
The effect of ICU diaries on **a** depression and **b** anxiety in patients

The study of Garrouste-Orgeas et al. [13] did not present the incidence of depression in patients. The point estimates of all remaining studies were consistent by showing a decrease in the risk of depression with the use of ICU diaries, but the confidence intervals crossed the null value. When pooling the results, a relative reduction of 69.0% in the risk of depression associated with ICU diaries was observed (Fig. 3).

#### Anxiety

Six studies investigated the effect of ICU diaries on anxiety diagnosis and severity of symptoms [9, 11–13, 28, 32]. Four of them are randomized controlled trials [9, 11–13]. As in the studies of depression, all studies used the same scale to evaluate and diagnose anxiety—the Hospital Anxiety and Depression Scale (HADS)—but different thresholds were used to dichotomize the variable (Additional file 2: Figure S2).

##### Severity of symptoms of anxiety measured by mean or median HADS

One RCT reported a decrease of anxiety symptoms in patients that received the ICU diary. Nielsen et al. [12] and Garrouste-Orgeas et al. [28] did not report HADS score as a continuous variable. Knowles and Tarrier [9] and Garrouste-Orgeas et al. [13] did not identify any difference in HADS scores between patients who received or not an ICU Diary. Fukuda et al. [32] found a decrease in HADS score (from 7.1 ± 3.8 to 5.7 ± 2.7 points, *p* = 0.011) only in patients with distorted memories (Additional file 2: Figure S2).

##### Anxiety diagnosis

The study of Garrouste-Orgeas et al. [13] did not present the incidence of anxiety in patients. Only one study showed a decrease of anxiety diagnosis for patients receiving an ICU diary [11]. When pooling all the results, there was no difference of anxiety between patients that received an ICU diary and those who did not (Fig. 3).

#### Quality of life

**Fig. 4:**
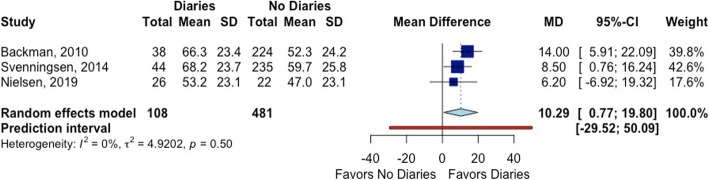
The effect of ICU diaries on patients’ quality of life (global health score of the SF-36)

Three studies evaluated the impact of ICU diaries on quality of life using the Modified Medical Outcomes Short Form (SF-36) [12, 27, 31]. Only one of them was a RCT, and it did not show a difference between ICU diary and no diary in any of the domains of the SF-36 at 3 months of follow-up [12]. In the Bäckman et al. study [27], patients who received a diary had a higher mean score for the global health (GH) and vitality (VT) domains of the SF-36 at 6 months. Svenningsen et al. [31] reported an improvement only in the GH subsection of the SF-36 at 6 months (mean values of other domains were not presented). We pooled only GH scores from SF-36 (Fig. 4).

### The effect of ICU diaries on outcomes of the patient’s relatives

Four studies evaluated the effect of ICU diaries on the family’s PTSD [12, 13, 28, 30]. One study reported no difference between the group that received or not the ICU diary [13]. The remaining three studies showed a decrease in PTSD incidence, but with a low precision of estimates. When pooling the results, there were no differences in the incidence of PTSD between those who received the ICU diary and those who did not.

**Fig. 5:**
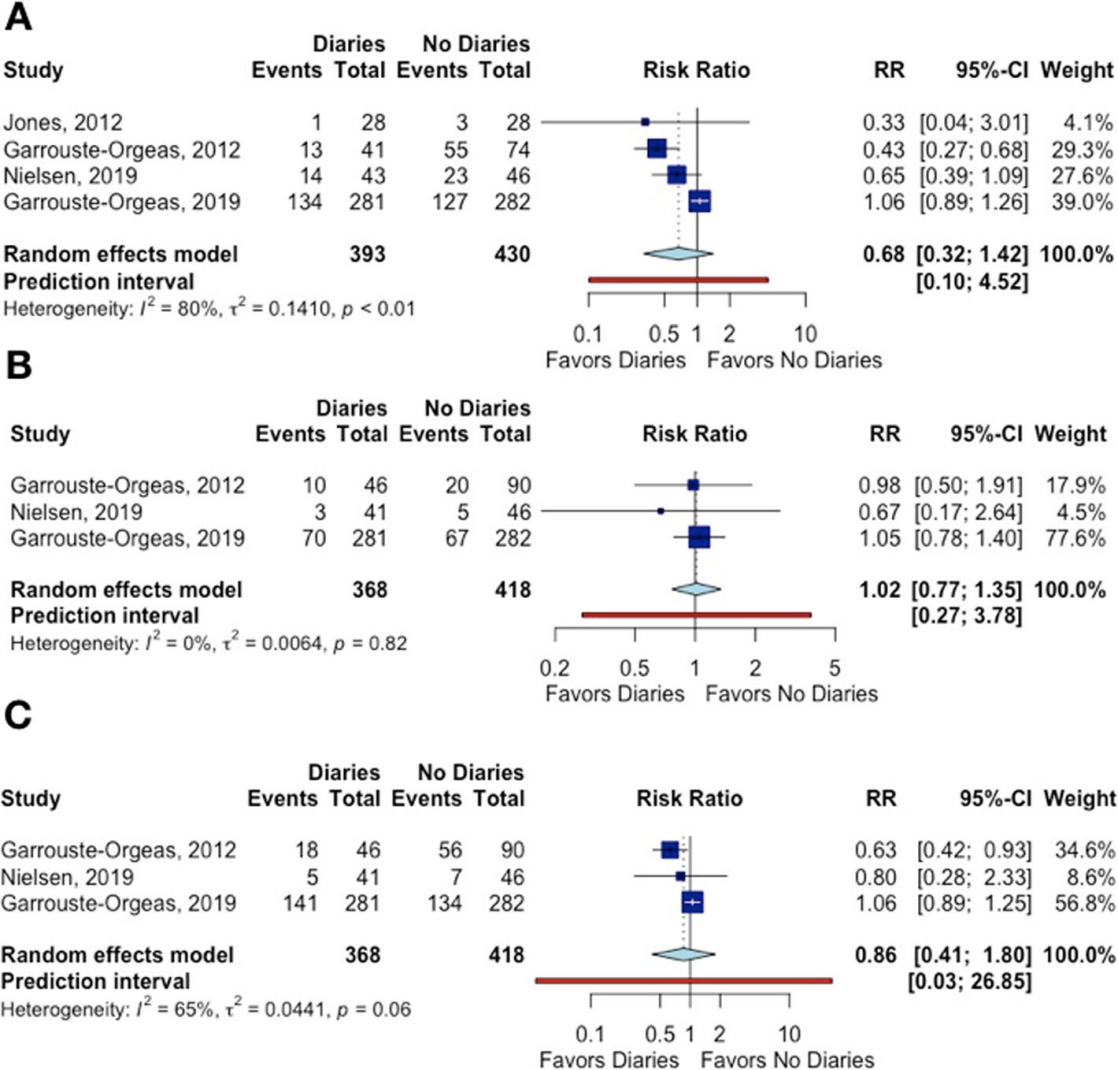
The effect of ICU diaries on **a** post-traumatic stress disorder, **b** depression, and **c** anxiety in patients’ relatives

Three studies investigated the impact of ICU diaries on depression and anxiety among relatives [12, 13, 28]. Only Garrouste-Orgeas et al. [28] reported an improvement in anxiety (Fig. 5).

### Patients’ impressions about receiving an ICU diary

Seven studies reported patients’ and relatives’ feedbacks about receiving and reading the ICU diary [8, 9, 11, 12, 26, 29, 32]. Mostly, patients felt that the diaries helped them to (1) connect their own memories to what actually happened during the ICU admission, (2) connect with their families as a way to confirm information presented on the diary or as a way to understand what they also have been through during ICU admission, and (3) improve perception of ICU care [32]. Also, the presence of photos was considered “good or very good,” leading to a greater understanding of what it looked like in the ICU and how ill they had been [29].

### Small-study effect

The studies that reported PTSD, anxiety, and depression outcomes for both patients and relatives were tested for small-study effect by the use of funnel plots. None of the funnel plots showed any evidence of a small-study effect. The *p* values for Egger tests were > 0.05 (Additional file 2: Figure S3 to S8).

## Discussion

The results of this systematic review and meta-analysis show that patients treated in intensive care units who received a diary had a lower risk of depression and better health-related quality of life measured by the global health domain of the SF-36 than those who did not receive a diary. On the other hand, there were no differences in PTSD and anxiety in patients, and the intensity of symptoms of depression and anxiety of patients who receive ICU diaries was similar to those who did not receive it. For the relatives of the patients, the results did not show improvement of psychological sequelae with the use of ICU diaries.

Since the beginning of the year 2000 [33], the use of ICU diaries has been extensively studied as a tool to enhance recovery after ICU admission [9, 12, 27–29, 32–47]. However, in the review for the present meta-analysis, we observed that only the minority of studies on ICU diaries evaluated the incidence of psychiatric diseases using validated scales as outcomes. A meta-analysis published in 2015 [48] based on the data of only three randomized trials [8, 9, 30] indicated that there was minimal evidence to support the effectiveness of ICU diary in improving psychological recovery after critical illness for patients, their caregivers, or family members. A more recent meta-analysis with studies published until 2017 included eight studies: the three trials [8, 9, 30] from the previous meta-analysis [48], two time series [28, 32], and three observational studies [27, 29, 31]. Similarly to the present meta-analysis, ICU diary was associated with lower symptoms of anxiety and depression, but not with post-traumatic stress syndrome [10]. Since then, four new studies have been published including three randomized controlled trials on the subject [11–13], which were included in the current meta-analysis.

The analysis of the present study was mainly based on quantitative data studies. However, we identified qualitative data studies that deserve mentioning. We found 21 qualitative studies about the perceptions of patients, families, and healthcare workers on writing and receiving ICU diaries [7, 33–47, 49–53]. Families of the patients who wrote and/or read the ICU thought that diaries were important to enhance access to and assimilation of medical information about the patient. They reported that the ICU diaries served as a channel of communication and approximation among family members in a tough moment of their lives and helped them to cope with overwhelming emotional experiences [40]. By writing and reading the diaries, family members could document their presence at the patient’s side, express their love and affection, confide their intimate feelings, and struggled to maintain hope. Reading the diary also made the relatives aware that the staff saw patients as a living human being, and the time devoted by healthcare providers to the diary was seen by the family as a sign of consideration, emotional involvement, and empathy [40]. These feelings were identified in other studies [34, 41, 42], and even families of deceased patients thought that the diary offered consolation and helped them to cope bereavement [34, 44].

For healthcare providers, writing the diary helped to perceive the human dimension to their work and to improve communication with the families as an adjunct to oral communication [38]. Staff members also considered the reading of the diary beneficial by allowing a deeper knowledge of the patients as a human being through the eyes of their loved ones and broadening the perception of how hard ICU care can be on family members [38, 43]. It should be noted, however, that staff members also reported strong emotions and concerns about intrusion into the patients’ and families’ privacies related to diary writing and reading [38].

While the present study offers additional evidence about the role of ICU diary on psychiatric outcomes for patients and their relatives, methodological limitations cannot be ignored. One such potential limitation is the different designs of the selected studies (observational, before-and-after, randomized controlled trials), which are susceptible to different sources of bias. To reduce the influence of bias in the results, specific analyses by study type were performed. Variation among the studies in sample size, in the time of intervention, in tools used to diagnose PTSD, in the threshold used for the psychiatric diagnosis, and in follow-up duration was observed. A random-effects method was used to take into account these variations among studies. Despite the expected variations, the results of the *I*^2^ statistic indicated a small heterogeneity in the association of ICU diary with some outcomes.

Another important consideration to be made refers to the recent study of Garrouste-Orgeas et al., the largest clinical trial on the topic so far [13]. This study was performed in 35 French ICUs, and they could not find any improvement in clinical outcomes with the use of the ICU diary. However, it is important to highlight that, despite the anticipation of a high mortality rate when calculating the sample size, a 50% rate of loss to follow-up may bias the association between the intervention and the outcome, as well as compromise the generalizability of the results.

Some strengths of the present systematic review also merit attention. By reviewing studies not included in a previously published meta-analysis, the present review expands the knowledge of the association between ICU diary and psychiatric outcomes in patients and their relatives [10]. In addition, we described the structure of diaries used in the studies, the reported workload associated with writing the diary, and the perception of the participants about receiving the diary (this information can be found in Additional file 2). In general, the patients considered the ICU diary an important tool to aid recovery and connection to their loved ones.

## Conclusions

The results of the present meta-analysis suggest that ICU diaries may reduce the risk of depression and the negative effects of intensive care treatment on the quality of life of the patients. Considering the methodological limitations, the results of the meta-analysis support a beneficial effect of the ICU diaries by mitigating the psychological sequelae that are common after ICU admission. Adequately powered randomized trials should be developed to provide stronger evidence about the potential beneficial effects of ICU diaries on the psychiatric outcomes of patients and their relatives.

## Supplementary information

**Additional file 1:** PRISMA Checklist. It contains the Preferred Reporting Items for Systematic Reviews and Meta-Analysis (PRISMA) Checklist

**Additional file 2:** Supplementary Material. Contains the search strategy, results and supplementary tables and figures in addition to the ones presented in the manuscript.

**Additional file 3:** R Script (statistical analysis). It contains the R script used for analyzing data and creating forest plots for the meta-analysis.

## Data Availability

The datasets used and/or analyzed during the current study are available from the corresponding author on reasonable request.

## Abbreviations

ICU: Intensive care unit
PTSD: Post-traumatic stress disorder
RCT: Randomized controlled trial
ROBINS-I: Risk Of Bias In Non-randomized Studies of Intervention
HADS: Hospital Anxiety and Depression Scale
PDEQ: Peritraumatic Dissociative Experiences Questionnaire
PTSS-14: Post-traumatic Stress Symptoms screening tool
SF-36: Modified Medical Outcomes Short Form
IES-R: Impact of Event Scale-Revised
ASDS: Acute Stress Disorder Scale
