# Supplementary material for: The impact of intensive care unit diaries on patients’ and relatives’ outcomes: a systematic review and meta-analysis
Source: Crit Care. 2019 Dec 16;23:411. doi: 10.1186/s13054-019-2678-0 (PMC6916011; doi:10.1186/s13054-019-2678-0)
Supplement: Supplementary file 2 — Additional file 2: Supplementary Material. Contains the search strategy, results and supplementary tables and figures in addition to the ones presented in the manuscript. [file 13054_2019_2678_MOESM2_ESM.pdf]

Additional File 2

The impact of ICU Diaries on patients' and relatives' outcomes: a Systematic Review and Meta-analysis

Bruna Brandao Barreto M.D., MSc; Mariana Luz M.D.; Marcos Nogueira de Oliveira Rios M.D.; Antonio Alberto Lopes M.D., PhD; Dimitri Gusmao-Flores M.D., PhD

SEARCH STRATEGY

Pubmed:

((((((((((("critical care") OR "critical illness") OR "Intensive Care Units") OR "critical illnesses") OR "Critically Ill") OR "intensive care") OR "ICU") OR "intensive care department") OR "Intensive Care Unit") OR "intensive care units") OR "high-dependency care unit") OR "Critical Care Unit")) AND ((((((((“Diaries”) OR “ICU Diaries”) OR "Intensive Care Diaries") OR "Critical Care Diaries") OR "Diary") OR "Critical Care Diary") OR "Intensive Care Diary") OR “ICU Diary”)

OVID:

(critical care OR critical illness OR Intensive Care Units OR critical illnesses OR Critically Ill OR intensive care OR ICU OR intensive care department OR Intensive Care Unit OR intensive care units OR Critical Care Unit) AND (Diaries OR ICU Diaries OR ICU Journal OR Intensive Care Diaries OR Critical Care Diaries OR Diary OR Critical Care Diary OR Intensive Care Diary OR ICU Diary)

Embase:

('intensive care' OR 'intensive care unit' OR 'critically ill patient' OR 'critical illness') AND ('icu diaries' OR diaries OR diary OR 'icu diary')

EBSCO host + PsycINFO:

(critical care OR critical illness OR Intensive Care Units OR critical illnesses OR Critically Ill OR intensive care OR ICU OR intensive care department OR Intensive Care Unit OR intensive care units OR Critical Care Unit) AND (Diaries OR ICU Diaries OR Intensive Care Diaries OR Critical Care Diaries OR Diary OR Critical Care Diary OR Intensive Care Diary OR ICU Diary)

RESULTS

Measuring agreement

- Study selection: Disagreement was present in the evaluation of two studies (kappa 0.913 – excellent agreement). This disagreement was caused by an oversight on the part of one of the authors, and it was quickly solved (1 study was included and the other was excluded at the end of the analysis).

- Data extraction: There was no disagreement on data extraction.

- Risk of bias assessment: There was no disagreement on the final judgments of risk of bias. There was a minor disagreement on one domain in two different studies, but this disagreement did not change the final risk of bias (both studies were already classified as "high risk")

Writing the ICU Diary

Two of the eleven studies did not provide a brief description of the intervention[1,2]. However, Akerman et al reported that the ICUs had guidelines for keeping diaries during the patient's ICU stay. Six studies reported that diary's entries were written by ICU staff and family members or friends, if they wanted to [3-8]. In two of them, entries were written by staff members [9,10], and one of them was written only by the relatives[11]. Most studies (8/11) described the following diary structure:

1) Summary: a brief summary of patients' illness and reasons leading to admission was usually included, along with initial events on the ICU and the current state of the illness at the day when the diary has begun to be written.
2) Language: most studies reinforced the importance of everyday language improve patients understanding of his/her ICU trajectory.
3) What to write: Common themes included providing a daily update about illness evolution; patients interactions with family, visitors and health care providers; family's feelings about the current situation; world and family events that would interest the patients; supporting comments and vows of recovery.
4) What not to write: confidential matters that would not be shared among ICU staff and friends; negative reactions and personal opinions.
5) Photos: most of the studies allowed photographs to be taken from patients (after consent of the patient himself or relatives), especially during interaction with family and health care providers. Photos were also taken in turning points during recovery and rehabilitation. There was a frequent concern of only attaching photos to the diary after patients' consent [7,8,11]. In some studies, drawing of children and family members were also allowed.

Kredentser et al[4] reported that families and friends wrote a mean of 1.7 entries per day, while staff members wrote a mean of 1.5 entries. Both family and health care providers reported a low burden when writing a diary, with 48% of entries taking less than 5 minutes.

Other Outcomes

Besides the previously detailed outcomes, the included studies also evaluated: Acute Stress Disorder[9], Dissociative Experiences[5], Physical and Mental Impairment (using the 3-set-4P questionnaire)[2] and recollection of patients memories of ICU after ICU discharge using the ICU Memory Tool. Fukuda et al[9] used the Acute Stress Disorder Scale (ASDS), a tool to screen for acute stress disorder consisting of a 19-item questionnaire answered by the individual. The decrease in acute distress symptoms was only seen in patients with distorted memories. In the study of Garrouste-Orgeas et al[5], the dissociative experiences were evaluated with the Peritraumatic Dissociative Experiences Questionnaire. There was no difference at the perception of a traumatic experience between patients who received and who did not receive an ICU Diary.

The study of Akerman et al reported more problems in Change of Appearance, Mood and Memory at 12 months in the group of patients who received an ICU Diary, measured with the 3-set-4P questionnaire[2]. However, it is important to observe that the baseline characteristics of the groups were severely unbalanced. Patients who received an ICU Diary spent more hours under mechanical ventilation (median 73,5h versus 0h, p-value <0.001), more days in the ICU (median 6 days versus 2 days, p-value <0.001) and they were more severely ill (mean SAPS III 58.4 versus 54, p-value <0.002) with a greater mortality risk (Estimated Mortality Risk 36.3 versus 28, p-value < 0,001).

The study of Garrouste-Orgeas et al[12] evaluated the recollection of patients' memories of ICU stay 3 months after ICU discharge using the ICU Memory Tool. There was no difference between the memories of patients who received the ICU diary and those who did not. In the intervention group, 106 of 158 patients (67.1%) experienced delusional memories versus 108 of 161 (67.1%) in the control group (Risk difference 0% (−11 to 11), p-value >.99).

Patients' impressions about receiving an ICU Diary

Seven studies reported patients and relatives feedbacks about receiving and reading the ICU Diary[2,4,6,7,9-11]. Fukuda et al[9] interviewed 17 patients in order to identify what meant for them to receive an ICU diary. Mostly, patients felt that the diaries helped them to: 1) connect their own memories to what it actually happened during the ICU admission; 2) connect with their families as a way to confirm information presented on the diary or as a way to understand what they also have been through during ICU admission and 3) to improve perception of ICU care.

Kredentser et al[4] reported on their study that most of the patients read their diaries three to five times after the initial meeting with the study nurse (53% of patients, 12/22) within 90 days after ICU discharge. Many of them reported reading their diary with family and friends (81% - 21/26). Patients reported that the most helpful aspect of the diary intervention was reviewing the diary with study personnel at the time of receipt[4].

Despite the negative outcome identified with the 3-set-4P questionnaire, patients included in Akerman et al also presented a positive attitude toward receiving the diary[2], which was perceived as being valuable during the rehabilitation process (86% of patients at 6 months – 114/133). Patients reported that the diary has given a greater understanding of the ICU admission (80% - 106/132) and it has helped to fill in memory gaps (80% - 106/132).

In the study of Glimelius Petersson et al[6], only 2 out of 40 patients did not read the diary given to them, but both of them reported that their relatives read it. The others reported the same idea that the diary "complements other memories", "helps you understand that there are many who care about you", and 60% reported that the diary helped them to understand the time spent in the ICU. This study specifically evaluated the presence of photos, and they were considered "good or very good", leading to greater understanding of what it looked like in the ICU and how ill they had been.

Jones et al[7] reported that patients' feedback about the diaries were also positive. In their study, patients read the diary a median of 3 times (0 to 20 range) and most of them shared the diaries with family (100%) and friends (36%). Patients felt that reading the diary was more helpful that the meeting with the study nurse at follow up.

Nielsen et al[11] reported twelve feedbacks from relative and three from patients. All patients stated that the diary had helped them "understand and process what happened together with a close relative". Only one relative (1/12) presented a negative attitude toward the diary stating that it could be difficult to read it again. Nine (9/12) were very positive, describing the diary as a place to unload emotions, and a help to remember and understand the ICU stay. Two (2/12) were neutral.

Knowles et al[10] reported that the qualitative feedback from participants about the diaries were very positive, with some regretting that diary was written only at the ICU. They also reported that receiving a diary improved patients perception of ICU care, and in their study there was a consensus that increasing the level of detail in the diary improved its quality.

Reference

1. **Svenningsen H, Tonnesen EK, Videbech P, Frydenberg M, Christensen D, Egerod I. Intensive care delirium - effect on memories and health-related quality of life - a follow-up study. *Journal of clinical nursing.* Mar 2014;23(5-6):634-644.**

2. Akerman E, Langius-Eklof A. The impact of follow-up visits and diaries on patient outcome after discharge from intensive care: A descriptive and explorative study. *Intensive & critical care nursing.* Dec 2018;49:14-20.
3. Backman CG, Orwelius L, Sjoberg F, Fredrikson M, Walther SM. Long-term effect of the ICU-diary concept on quality of life after critical illness. *Acta anaesthesiologica Scandinavica.* Jul 2010;54(6):736-743.
4. Kredentser MS, Blouw M, Marten N, et al. Preventing Posttraumatic Stress in ICU Survivors: A Single-Center Pilot Randomized Controlled Trial of ICU Diaries and Psychoeducation. *Critical care medicine.* Dec 2018;46(12):1914-1922.
5. Garrouste-Orgeas M, Coquet I, Perier A, et al. Impact of an intensive care unit diary on psychological distress in patients and relatives*. *Critical care medicine.* Jul 2012;40(7):2033-2040.
6. Glimelius Petersson C, Ringdal M, Apelqvist G, Bergbom I. Diaries and memories following an ICU stay: a 2-month follow-up study. *Nursing in critical care.* Nov 2018;23(6):299-307.
7. Jones C, Backman C, Capuzzo M, et al. Intensive care diaries reduce new onset post traumatic stress disorder following critical illness: a randomised, controlled trial. *Critical care.* 2010;14(5):R168.
8. Jones C, Backman C, Griffiths RD. Intensive care diaries and relatives' symptoms of posttraumatic stress disorder after critical illness: a pilot study. *American journal of critical care : an official publication, American Association of Critical-Care Nurses.* May 2012;21(3):172-176.
9. Fukuda T, Inoue T, Kinoshita Y, Yukawa T. Effectiveness of ICU Diaries: Improving "Distorted Memories" Encountered during ICU Admission. *Open Journal of Nursing.* April 2015 2015;5:313-324.
10. Knowles RE, Tarrier N. Evaluation of the effect of prospective patient diaries on emotional well-being in intensive care unit survivors: a randomized controlled trial. *Critical care medicine.* Jan 2009;37(1):184-191.
11. Nielsen AH, Angel S, Egerod I, Lund TH, Renberg M, Hansen TB. The effect of family-authored diaries on posttraumatic stress disorder in intensive care unit patients and their relatives: A randomised controlled trial (DRIP-study). *Australian critical care : official journal of the Confederation of Australian Critical Care Nurses.* Feb 19 2019.
12. Garrouste-Orgeas M, Flahault C, Vinatier I, et al. Effect of an ICU Diary on Posttraumatic Stress Disorder Symptoms Among Patients Receiving Mechanical Ventilation: A Randomized Clinical Trial. *Jama.* Jul 16 2019;322(3):229-239.

FIGURES

FIGURE S1. Detailed quality assessment with Cochrane Risk of Bias Tool for RCTs and Before/After studies

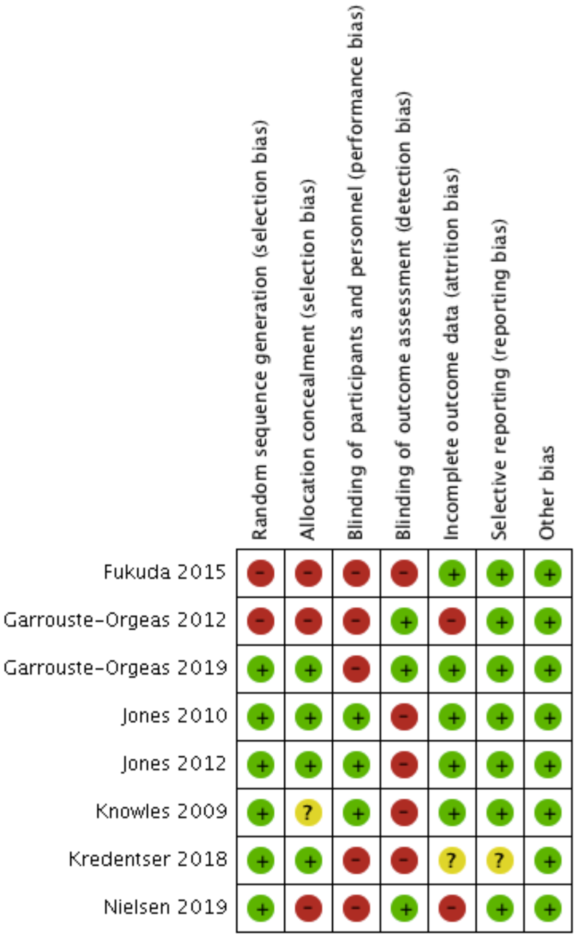

FIGURE S2. The effect of ICU Diaries on: (A) intensity of depressive symptoms and (B) intensity of anxiety symptoms in patients

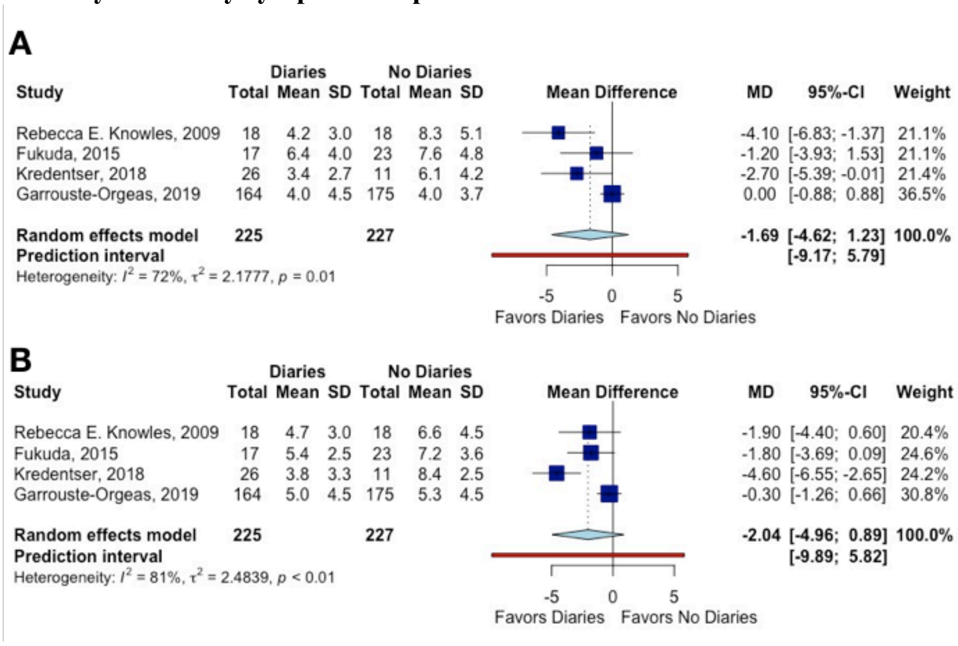

FIGURE S3. (A) Funnel plot and (B) Linear regression test of funnel plot asymmetry of studies evaluating the effect of ICU Diaries on PTSD (p-value = 0.949)

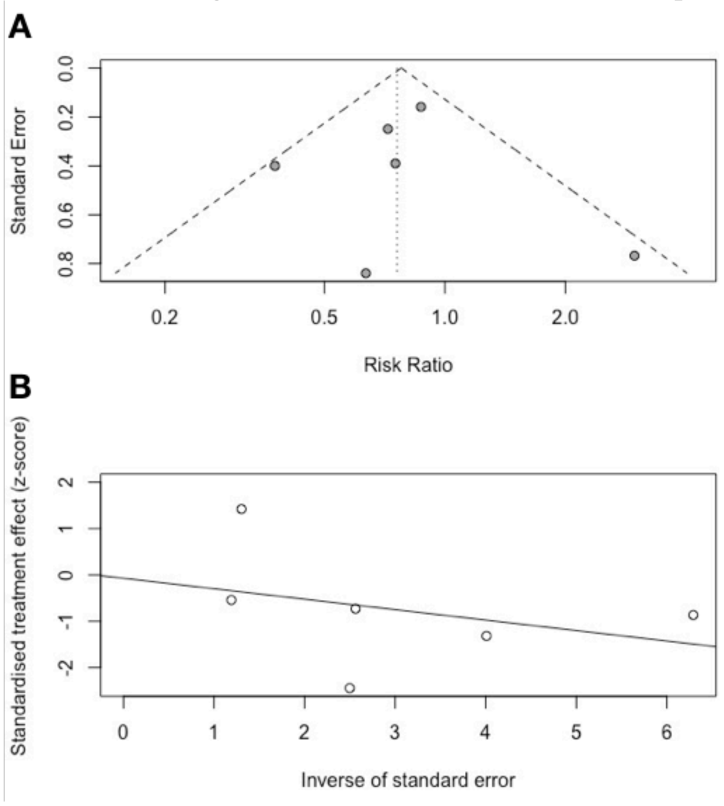

FIGURE S4. (A) Funnel plot and (B) Linear regression test of funnel plot asymmetry of studies evaluating the effect of ICU Diaries on Depression (p-value = 0.5485)

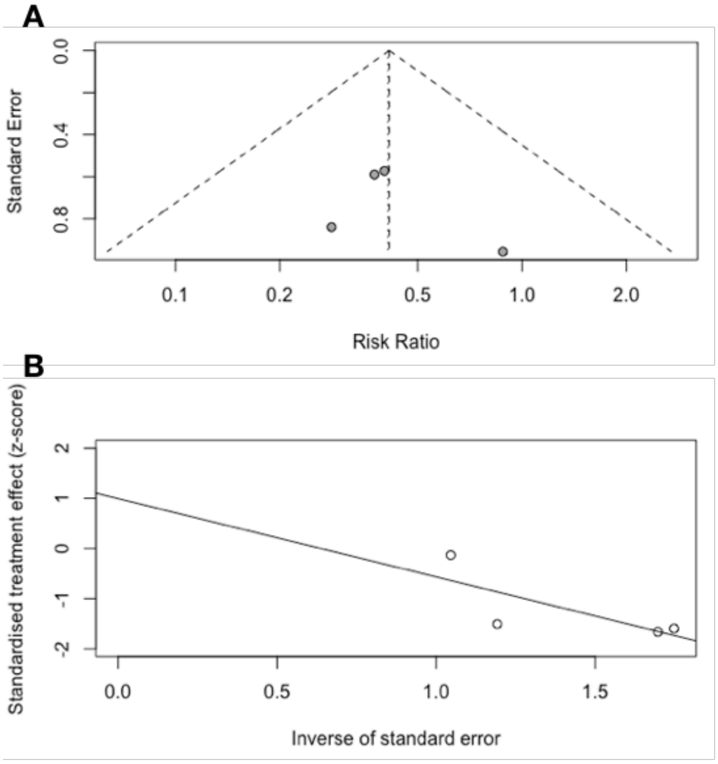

FIGURE S5. (A) Funnel plot and (B) Linear regression test of funnel plot asymmetry of studies evaluating the effect of ICU Diaries on Anxiety (p-value = 0.6761)

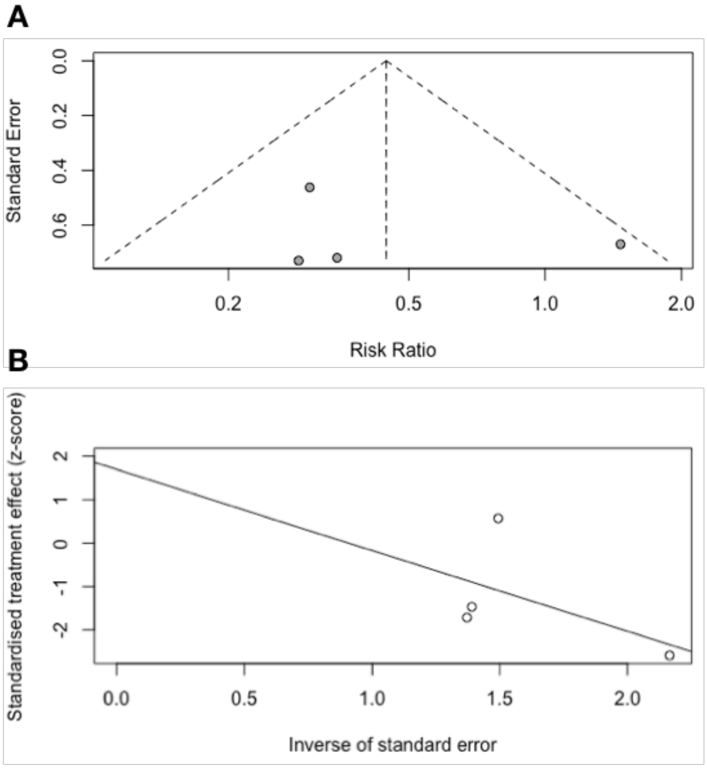

FIGURE S6. (A) Funnel plot and (B) Linear regression test of funnel plot asymmetry of studies evaluating the effect of ICU Diaries on PTSD in relatives (p-value = 0.2293)

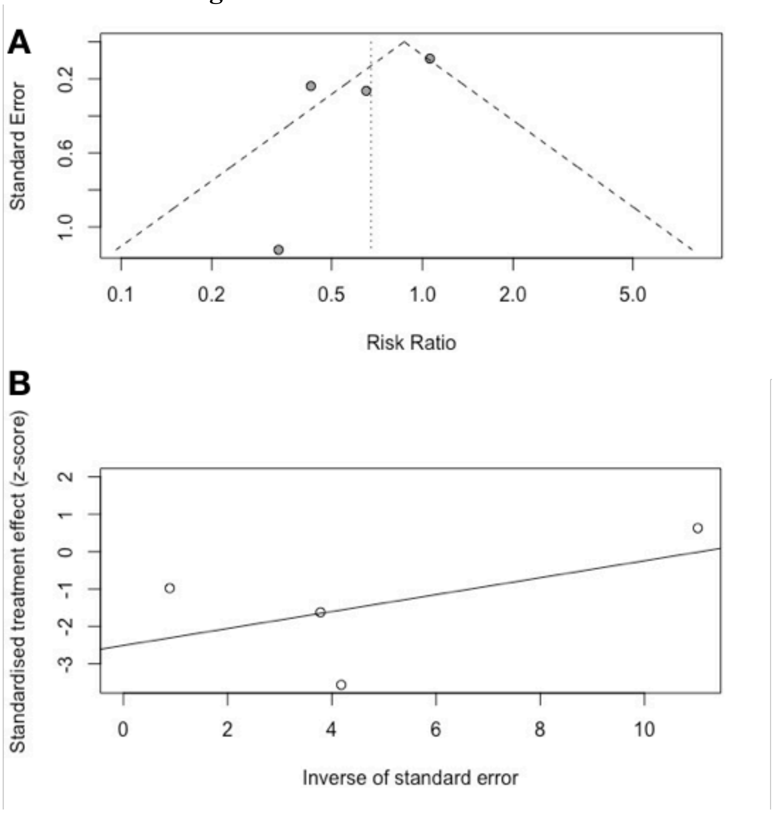

FIGURE S7. (A) Funnel plot and (B) Linear regression test of funnel plot asymmetry of studies evaluating the effect of ICU Diaries on Depression in relatives (p-value = 0.2047)

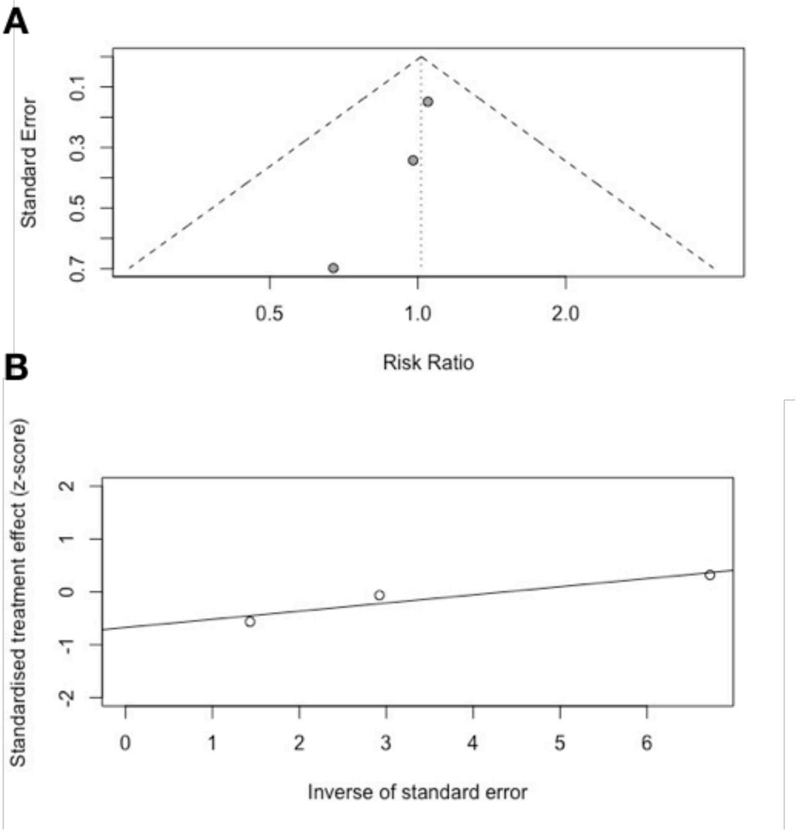

FIGURE S8. (A) Funnel plot and (B) Linear regression test of funnel plot asymmetry of studies evaluating the effect of ICU Diaries on Anxiety in relatives (p-value = 0.5354)

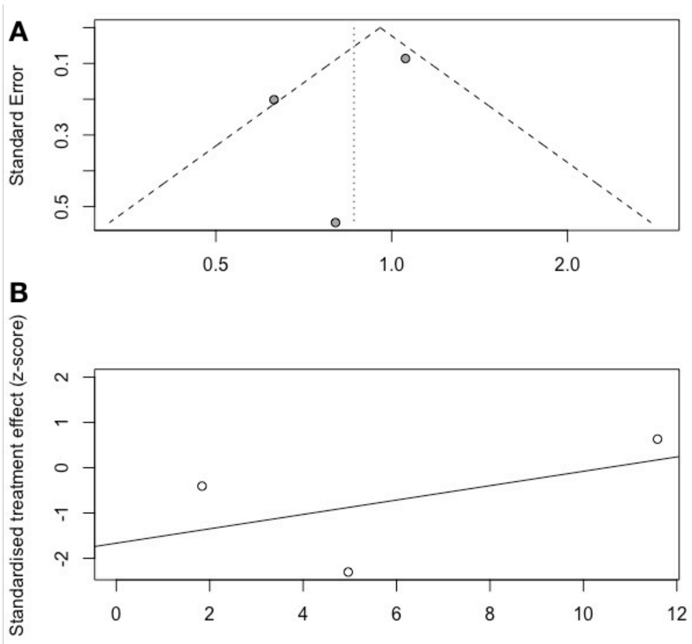

TABLE S1. Scales and Cutoffs used in each study for diagnosing PTSD or severe PTSD symptoms

Study	Scale	Threshold
Nielsen et al, 2019	PTSS-14	PTSS-14 ≥ 31
Maia S. Kredentser et al., 2018	IES-R	IES-R mean score ≥ 1.6
Christina Jones, 2012	PTSS-14	PTSS-14 ≥ 45
Garrouste-Orgeas, 2012	IES-R	IES-R mean score ≥ 22
Garrouste-Orgeas, 2019	IES-R	IES-R mean score ≥ 22
Cecilia Glimelius Petersson, 2015.	PTSS-14	PTSS-14 ≥ 45

TABLE S2. Scales and Cutoffs used in each study for diagnosing Depression and Anxiety

Study	Scale	Threshold
Nielsen et al, 2019	HADS	HADS ≥ 11
Garrouste-Orgeas, 2019	HADS	HADS ≥ 8
Maia S. Kredentser et al., 2018	HADS	HADS ≥ 8
Garrouste-Orgeas, 2015	HADS	HADS ≥ 8
Knowles, 2009	HADS	HADS ≥ 8

Table S3: Studies, sample included and lost follow-up.

Author / Year	Patients and Relatives included / Lost follow-up (n)
Nielsen et al, 2019	• Usual Care: 60 relatives and 39 patients / Lost follow-up: 13 relatives and 17 patients<br>• ICU Diaries: 56 relatives and 36 patients / Lost follow-up: 12 relatives and 10 patients
Maia S. Kredentser et al., 2018	• Usual Care: 14 patients / Lost follow-up: 8<br>• ICU Diaries: 15 patients / Lost follow-up: 2<br>• Psychoeducation: 14 / Lost follow-up: 9<br>• ICU Diaries + Psychoeducation: 15 / Lost follow-up: 2
Christina Jones et al., 2012	• ICU Diaries: 18 relatives / Lost follow-up:3<br>• Usual Care: 18 relatives / Lost follow-up: 3
Christina Jones et al., 2010	• ICU Diaries: 175 patients / Lost Follow-up: 15<br>• Usual Care: 177 / Lost Follow-up: 15
Rebecca E. Knowles et al., 2009	• ICU Diaries: 18 patients / Lost Follow-up: 0<br>• Usual Care: 18 / Lost Follow-up: 0
Tomohide Fukuda et al., 2015	• Usual Care: 177 / Lost Follow-up: 15
Maité Garrouste-Orgeas et al., 2012	• Prediary: 48 relatives and patients / Lost Follow-up 3 months: 00 relatives and 27 patients / Lost Follow-up 12 months: 8 relatives and 31 patients<br>• Diary: 49 relatives and patients / Lost Follow-up 3 months: 3 relatives and 20 patients / Lost Follow-up 12 months: 8 relatives and 29 patients<br>• Postdiary: 46 relatives and patients / Lost Follow-up 3 months: 4 relatives and 34 patients / Lost Follow-up 12 months: 12 relatives and 27 patients.
Maité Garrouste-Orgeas et al., 2019	• ICU Diaries: 332 patients and 332 relatives / Lost Follow-up: 168 patients and 51 relatives<br>• Usual Care: 325 patients and 325 relatives/ Lost Follow-up: 150 patients and 43 relatives
Eva Åkerman et al., 2018	• 441 patients included / Lost Follow-up: 22
Cecilia G. Petersson et al., 2015	• Usual Care: 44 patients / Lost Follow-up: 10<br>• ICU Diaries: 52 patients / Lost Follow-up: 12
Helle Svenningsen et al., 2014	• 360 patients included / Lost Follow-up: 81
C. G. Backman et al., 2010	• No diary group: 459 patients / Lost Follow-up: 235<br>• Diary group: 40 patients / Lost Follow-up: 02
