# Supplementary material for: The impact of intensive care unit diaries on patients’ and relatives’ outcomes: a systematic review and meta-analysis
Source: Crit Care. 2019 Dec 16;23:411. doi: 10.1186/s13054-019-2678-0 (PMC6916011; doi:10.1186/s13054-019-2678-0)
Supplement: Supplementary file 3 — Additional file 3: R Script (statistical analysis). It contains the R script used for analyzing data and creating forest plots for the meta-analysis. [file 13054_2019_2678_MOESM3_ESM.pdf]

ICU Diaries - MA

Bruna Brandão, Dimitri Gusmao-Flores

11/07/2019

PATIENTS

TEPT

By study type

```
library(readxl)
MA_Diários <- read_excel("MA - Diários.xlsx")
library(meta)
library(metafor)
#library(metafor)
#http://www.metafor-
project.org/doku.php/plots:forest_plot_with_subgroups


TEPT_type <- metabin(event.e, n.e, event.c, n.c, studlab = Study,
data=MA_Diários,  hakn = TRUE, method.tau = "SJ", comb.fixed= F,
prediction = T, byvar = MA_Diários$Type, label.e= "Diaries", label.c=
"No Diaries", label.left= "Favors Diaries", label.right= "Favors No
Diaries")
forest (TEPT_type, col.diamond.random="lightblue",
col.square="darkblue", col.square.lines="darkblue", just = "center")
```

Depression

By study type

```
library(readxl)
MA_Diários <- read_excel("MA - Diários.xlsx")
library(meta)
library(metafor)
#library(metafor)
#http://www.metafor-
project.org/doku.php/plots:forest_plot_with_subgroups
Depression_type <- metabin(event.e, n.e, event.c, n.c, studlab = Study,
data=MA_Diários,  hakn = TRUE, comb.fixed= F, method.tau = "SJ",
prediction = T, byvar = MA_Diários$Type, label.e= "Diaries", label.c=
"No Diaries", label.left= "Favors Diaries", label.right= "Favors No
Diaries")
forest (Depression_type, col.diamond.random="lightblue",
col.square="darkblue", col.square.lines="darkblue", just= "center")
```

Intensity of Depressive symptoms

```
library(readxl)
MA_Diários <- read_excel("MA - Diários.xlsx")
library(meta)
library(metafor)
#library(metafor)
#http://www.metafor-
project.org/doku.php/plots:forest_plot_with_subgroups
MA_Diários$mean.c= as.numeric(MA_Diários$mean.c)
MA_Diários$mean.e= as.numeric(MA_Diários$mean.e)
MA_Diários$sd.c= as.numeric(MA_Diários$sd.c)
MA_Diários$sd.e= as.numeric(MA_Diários$sd.e)

Dep_symp=metacont(n.e, round(mean.e,1), round(sd.e,1), n.c,
round(mean.c,1), round(sd.c,1), studlab= Study, data=MA_Diários, hakn =
T, method.tau = "SJ", prediction = T, comb.fixed= F, label.e =
"Diaries", label.c = "No Diaries", label.left = "Favors Diaries",
label.right = "Favors No Diaries")
forest(Dep_symp, col.diamond.random="lightblue", col.square="darkblue",
col.square.lines="darkblue", just = "center", digits.sd=1, digits.me=1)
```

Anxiety

By study type

```
library(readxl)
MA_Diários <- read_excel("MA - Diários.xlsx")
library(meta)
library(metafor)
#library(metafor)
#http://www.metafor-
project.org/doku.php/plots:forest_plot_with_subgroups
Anxiety_type <- metabin(event.e, n.e, event.c, n.c, studlab = Study,
data=MA_Diários,  hakn = TRUE, method.tau = "SJ", prediction = T,
comb.fixed= F, byvar = MA_Diários$Type, label.e= "Diaries", label.c=
"No Diaries", label.left= "Favors Diaries", label.right= "Favors No
Diaries")
forest (Anxiety_type, col.diamond.random="lightblue",
col.square="darkblue", col.square.lines="darkblue")
```

Intensity of Anxiety Symptoms

```
library(readxl)
MA_Diários <- read_excel("MA - Diários.xlsx")
library(meta)
library(metafor)
#library(metafor)
#http://www.metafor-
project.org/doku.php/plots:forest_plot_with_subgroups
MA_Diários$mean.c= as.numeric(MA_Diários$mean.c)
MA_Diários$mean.e= as.numeric(MA_Diários$mean.e)
```

```
MA_Diários$sd.c= as.numeric(MA_Diários$sd.c)
MA_Diários$sd.e= as.numeric(MA_Diários$sd.e)

Anx_symp=metacont(n.e, round(mean.e,1), round(sd.e,1), n.c,
round(mean.c,1), round(sd.c,1), studlab= Study, data=MA_Diários, hakn =
T, method.tau = "SJ", prediction = T, comb.fixed= F, label.e =
"Diaries", label.c = "No Diaries", label.left = "Favors Diaries",
label.right = "Favors No Diaries")
forest(Anx_symp, col.diamond.random="lightblue", col.square="darkblue",
col.square.lines="darkblue", just = "center", digits.sd=1, digits.me=1)
```

SF-36 (Global Health)

```
library(readxl)
MA_Diários <- read_excel("MA - Diários.xlsx")
library(meta)
library(metafor)
#library(metafor)
#http://www.metafor-
project.org/doku.php/plots:forest_plot_with_subgroups
MA_Diários$mean.c= as.numeric(MA_Diários$mean.c)
MA_Diários$mean.e= as.numeric(MA_Diários$mean.e)
MA_Diários$sd.c= as.numeric(MA_Diários$sd.c)
MA_Diários$sd.e= as.numeric(MA_Diários$sd.e)

SF36=metacontmetacont(n.e, round(mean.e,1), round(sd.e,1), n.c,
round(mean.c,1), round(sd.c,1), studlab= Study, data=MA_Diários, hakn =
T, method.tau = "SJ", prediction = T, comb.fixed= F, label.e =
"Diaries", label.c = "No Diaries", label.left = "Favors No Diaries",
label.right = "Favors Diaries")
forest(SF36, col.diamond.random="lightblue", col.square="darkblue",
col.square.lines="darkblue", just = "center", digits.sd=1, digits.me=1)
```

RELATIVES

TEPT

```
library(readxl)
MA_Diários <- read_excel("MA - Diários.xlsx")
library(meta)
library(metafor)
#library(metafor)
#http://www.metafor-
project.org/doku.php/plots:forest_plot_with_subgroups
Relatives_TEPT <- metabin(event.e, n.e, event.c, n.c, studlab = Study,
data=MA_Diários,  hakn = TRUE, method.tau = "SJ", prediction = T,
comb.fixed= F, label.e= "Diaries", label.c= "No Diaries", label.left=
"Favors Diaries", label.right= "Favors No Diaries")
forest (Relatives_TEPT)
```

Depression

```
library(readxl)
MA_Diários <- read_excel("MA - Diários.xlsx")
library(meta)
library(metafor)
#library(metafor)
#http://www.metafor-
project.org/doku.php/plots:forest_plot_with_subgroups
Relatives_Dep <- metabin(event.e, n.e, event.c, n.c, studlab = Study,
data=MA_Diários,  hakn = TRUE, method.tau = "SJ", prediction = T,
comb.fixed= F, label.e= "Diaries", label.c= "No Diaries", label.left=
"Favors Diaries", label.right= "Favors No Diaries")
forest (Relatives_Dep)
```

Anxiety

```
library(readxl)
MA_Diários <- read_excel("MA - Diários.xlsx")
library(meta)
library(metafor)
#library(metafor)
#http://www.metafor-
project.org/doku.php/plots:forest_plot_with_subgroups
Relatives_Anx <- metabin(event.e, n.e, event.c, n.c, studlab = Study,
data=MA_Diários,  hakn = TRUE, method.tau = "SJ", prediction = T,
comb.fixed= F, label.e= "Diaries", label.c= "No Diaries", label.left=
"Favors Diaries", label.right= "Favors No Diaries")
forest (Relatives_Anx)
```
